# Establishing the Substantive Interpretation of the GFP by Considering Evidence from Research on Personality Disorders and Animal Personality

**DOI:** 10.3389/fpsyg.2017.01771

**Published:** 2017-10-09

**Authors:** Michael P. Hengartner, Dimitri van der Linden, Curtis S. Dunkel

**Affiliations:** ^1^Department of Applied Psychology, Zurich University of Applied Sciences, Zurich, Switzerland; ^2^Department of Psychology, Education, and Child Studies, Erasmus University Rotterdam, Rotterdam, Netherlands; ^3^Department of Psychology, Western Illinois University, Macomb, IL, United States

**Keywords:** general factor of personality, social effectiveness, life history theory, animal personality, ethology, evolutionary biology, personality disorders, personality pathology

In research on individual differences, various structural models aim at providing a comprehensive description of personality. These models assume multiple, mostly independent personality dimensions. More recently, the so-called General Factor of Personality (GFP) has become a proliferous, but contentious, topic. The notion of the GFP is based on the observations that personality dimensions are not independent, but in fact show consistent inter-correlations, leading to a relevant proportion of shared variance among them (Figueredo et al., 2006). The GFP seems to capture the socially desirable ends of personality scales, and, in terms of the Big Five model, high-GFP individuals score relatively high on openness, conscientiousness, extraversion (mainly the sociability-facet), agreeableness, and emotional stability (Rushton and Irwing, 2009; van der Linden et al., 2010a). Some authors have suggested that the GFP simply reflects methodological artifacts (Ashton et al., 2009; Backstrom et al., 2009; Hopwood et al., 2011b; Pettersson et al., 2012). However, much of this criticism has been addressed (Rushton and Erdle, 2010; Loehlin, 2012; Dunkel and van der Linden, 2014; van der Linden et al., 2014a). The objective of the present work is not to reiterate these issues, as they have been discussed extensively elsewhere (Irwing, 2013; van der Linden et al., 2016). Instead, we contend that criticism mostly offered within the specialty of personality psychology misses the bigger picture. More specific, evidence in favor of the GFP as a substantive and theoretically coherent construct has been provided in other research fields long before it became a contentious issue in personality psychology. Here we introduce two lines of evidence that may further corroborate the substantive interpretation of the GFP, specifically, findings from personality pathology as well as from animal personality. Looking at the GFP from a different perspective may help to overcome the current debates within personality psychology. In the following we will first briefly introduce work on the GFP and its theoretical foundation as social effectiveness. Afterwards we outline research from psychiatric nosology and animal ecology and discuss these in context.

## The GFP reflecting social effectiveness

Although most scholars would agree that a GFP can be identified in every assessment of personality dimensions, there is less consensus about the interpretation of this factor. Yet, one of its currently leading substantive interpretations is that the GFP largely reflects social effectiveness (Rushton et al., 2008; Loehlin, 2012; van der Linden et al., 2016). That is, high-GFP individuals seem to be characterized by a motivation to behave in socially desirable ways and by the tendency and ability to do so. As such, they would more often get along or get ahead socially which may enhance the probability of reaching their goals, such as, securing a desirable partner and easing access to socio-economic resources. A growing body of research supports this notion. First, measures of GFP have been found to overlap considerably with other constructs indicating social effective behavior (Dunkel and van der Linden, 2014). In a large meta-analysis, van der Linden et al. (2017) showed that the GFP has almost complete overlap (ρ = 0.88) with trait emotional intelligence and moderate associations (ρ = 0.28) with ability emotional intelligence. A second line of evidence shows that the GFP relates to various criteria of social success, including peer-rated likeability and popularity and higher ratings on several objective socio-economic performance indicators (van der Linden et al., 2010b, 2014a,b). Those findings are in accordance with the view that the GFP does not merely reflect bias, but instead arise because high-GFP individuals genuinely tend to behave in a more socially desirable manner, while low GFP scores would be associated with problematic social behavior, including violence, delinquency, and impulsive sensation-seeking. However, as detailed below, such predispositions may be evolutionary adaptive in specific ecologies (Figueredo et al., 2006; Ellis et al., 2012). For instance, in deviant subcultures such as inmates, violent offenders score consistently lower on the GFP (van der Linden et al., 2015).

## Evidence from personality disorder research

Psychiatric research has made strong and compelling arguments for a general factor of personality pathology long before personality psychologists became aware of and started debating the GFP (see reviews by Bornstein, 1998; Tyrer, 2005). With respect to personality disorders (PD), the general factor identified in those measures has received different terms, including severity of PD, general personality dysfunction, or general personality pathology. Though these notions may differ in scope and content, they are largely overlapping and converge on the notion that the severity of (social) impairments related to PD is the defining feature. Hence, for the sake of simplicity we will hereinafter use the term General Factor of Personality pathology (GFPP). The GFPP has been extensively validated across two decades in phenotypic research on the structure of PD traits (Tyrer and Johnson, 1996; Deary et al., 1998; Seivewright et al., 2004; Hopwood et al., 2011a; Morey et al., 2011; Wright et al., 2012; Hengartner et al., 2014a; Kim et al., 2014; Bastiaansen et al., 2016). There is also evidence from behavioral genetic studies supporting the notion of the GFPP as a substantive and valid construct (Livesley et al., 1998; Kendler et al., 2008; Reichborn-Kjennerud, 2010). Consistent with the social effectiveness hypothesis of the GFP, it has been posited that the GFPP mainly captures social dysfunction and interpersonal problems (Hengartner et al., 2015; Tyrer et al., 2015). Indeed, a growing body of literature, including both cross-sectional epidemiologic community studies and prospective longitudinal clinical studies, has consistently demonstrated that the GFPP relates to social functioning deficits (Yang et al., 2010; Hopwood et al., 2011a; Hengartner et al., 2014b; Conway et al., 2016; Wright et al., 2016). Finally, recent advances in psychiatric nosology, based on both phenotypic and genetic research, now provide strong evidence for a general factor of psychopathology, coined the *p* factor, at the apex of the hierarchical structure of psychopathology (Lahey et al., 2011, 2012; Laceulle et al., 2015; Pettersson et al., 2016). The *p* factor extends the GFPP by accounting for covariance between all psychiatric syndromes, and not exclusively between PD traits. The *p* factor shows meaningful associations with normative personality traits consistent with their GFP loadings, i.e., positive associations with neuroticism (or negative affectivity) and negative associations with agreeableness and conscientiousness (or effortful control; Tackett et al., 2013; Caspi et al., 2014; Castellanos-Ryan et al., 2016; Hankin et al., 2017). Nevertheless, to the best of our knowledge, a direct test of the association between the GFP, the GFPP and the *p* factor has not been provided yet, though we note that the GFP has been related to a general health factor that contained general psychopathology (Figueredo et al., 2007; Figueredo and Rushton, 2009). A promising avenue for future research would hence be to directly test correlations between the GFP, the GFPP, and the *p* factor. By putting these pieces together, researchers may further advance our understanding of the pervasive impact of personality on human health and social functioning (Ozer and Benet-Martinez, 2006; Smith and MacKenzie, 2006; Hengartner, 2015).

## Evidence from animal personality

Research in ethology and evolutionary biology has shown that inter-individual variation in personality traits can be observed within and between animal taxa (Gosling, 2001; Reale et al., 2007). These studies confirm that personality is not unique to human, but rather a universal biological phenomenon with important implications for ecology and evolution (Wolf and Weissing, 2012). Because personality is moderately heritable in humans and other animals (Bouchard and Loehlin, 2001; Dochtermann et al., 2015), it was further proposed that personality trait variation must be subjected to natural selection (Nettle, 2006; Penke et al., 2007). In the following we will introduce an evolutionary model of animal personality based on life history (LH) theory (Roff, 2002), as LH theory plays also a crucial role in models of human GFP (Figueredo et al., 2006; Rushton et al., 2008). LH theory posits that individuals must allocate finite resources such as time and energy to competing LH traits including reproduction, health maintenance, mating, and parenting. Because resources are inherently limited, investment toward one trait reduces investments toward the others. As a result, trade-offs between LH traits emerge giving rise to a broad superordinate factor of LH strategy (LHS) that encompasses the extreme poles of fast vs. slow. Individuals following a fast LHS tend to invest relatively many resources into reproductive efforts. A slow LH strategy on the other hand, is characterized by higher investments toward somatic efforts (Roff, 2002; Jones, 2011). As detailed by two evolutionary models, selection for fast vs. slow LHS results in correlated personality traits due to trade-offs between somatic and reproductive efforts (Stamps, 2007; Wolf et al., 2007). That is, individuals who invest heavily in early reproduction (fast LHS) must be bold and aggressive in order to outcompete rivals and attract mates, whereas individuals who delay reproduction (slow LHS) must be risk-averse, sociable, and docile, otherwise they would not survive long enough to benefit from future fitness returns (Hengartner, in press). Boldness-aggressiveness thus constitutes a behavioral syndrome, also termed proactive personality profile, whereas sociability-docility forms another syndrome, coined reactive personality profile (Groothuis and Carere, 2005; Koolhaas et al., 2010). Both syndromes are supposed to be extreme poles along a common GFP-like personality dimension that is co-adapted to LHS (Reale et al., 2010). That is, proactive personalities reflect low GFP scores (asocial, irritable, aggressive, and impulsive), while reactive personalities reflect high GFP scores (sociable, cautious, empathic, and self-controlled) (Hengartner, in press). Mounting evidence from different animal species now supports these personality profiles as correlates of LH traits (Biro and Stamps, 2008; Careau et al., 2009, 2010; Le Galliard et al., 2013; Niemela et al., 2013; Montiglio et al., 2014; Schuett et al., 2015; Binder et al., 2016; but see also Debecker et al., 2016). Noteworthy, Weiss et al. (2011) found no evidence for a GFP in the personality structure of chimpanzees, orang-utans, and rhesus macaques. However, their results were derived from a factor-analytic approach that neither included lower-order facets or primary items nor correlated trait-residuals. Research in humans has clearly shown that to successfully extract a GFP sometimes requires detailed structural modeling of lower-order facets and their interdependence (Rushton and Irwing, 2009). In correspondence, Latzman et al. (2014) extracted a hierarchical structure of personality in chimpanzees based on 43 primary items that was very similar to humans, including two higher-order factors corresponding to externalizing and internalizing personality. Since in humans externalizing and internalizing personality commonly load onto a GFP (Wright and Simms, 2014; Hengartner et al., 2017), these findings raise the possibility of a GFP in chimpanzees, too.

## Conclusions and outlook

In the present paper we argued that the GFP likely represents a substantive factor that can contribute to integrate the literature from various fields on individual differences, including normative personality, personality pathology, LH theory, and animal personality. The literature briefly reviewed suggests that it would appear misguided to refute the GFP as a methodological artifact. Instead, we propose that future research in personality psychology should put more emphasis on the phylogenetic and ontogenetic origin of the GFP as a measure of social effectiveness that aligns with LHS. In humans, the GFP has been shown to correlate strongly with LHS (Figueredo et al., 2007; Figueredo and Rushton, 2009; Dunkel et al., 2012), but unfortunately all these human studies relied exclusively on subjective self-report assessments of LHS (Figueredo et al., 2013). Linkage with animal personality as well as implementation of objective biological LH measures routinely applied in ethological research (e.g., age at first reproduction), would therefore considerably improve the validity of human GFP research. Moreover, linking the evolutionary origins of the GFP with psychiatric nosology may help to explain the pervasive socio-ecological impairments related to the GFPP (Hopwood et al., 2011a; Hengartner et al., 2014b; Wright et al., 2016). Finally, because it is supposed that both slow and fast LHS entail fitness benefits depending on contingent environmental demands (Ellis et al., 2009), it would be necessary to extend the social effectiveness hypothesis of the GFP by focusing on the adaptive solutions provided by both high and low GFP-scores (Figueredo et al., 2006; Sherman et al., 2013).

## Author contributions

MH drafted the manuscript. DvdL and CD participated in writing and critical revision of the manuscript. All authors approved the final version.

### Conflict of interest statement

The authors declare that the research was conducted in the absence of any commercial or financial relationships that could be construed as a potential conflict of interest.
